# Identification, Characterization and Expression Profiling of the RS Gene Family during the Withering Process of White Tea in the Tea Plant (*Camellia sinensis*) Reveal the Transcriptional Regulation of *CsRS8*

**DOI:** 10.3390/ijms24010202

**Published:** 2022-12-22

**Authors:** Tao Wang, Yiqing Wang, Jiamin Zhao, Jiumei Kong, Lingzhi Zhang, Siyu Qi, Jiajia Chen, Zhidan Chen, Wen Zeng, Weijiang Sun

**Affiliations:** 1 College of Horticulture, Fujian Agriculture and Forestry University, Fuzhou 350002, China; 2 Anxi College of Tea Science, Fujian Agriculture and Forestry University, Quanzhou 362000, China

**Keywords:** *Camellia sinensis*, raffinose synthase, white tea, expression pattern, CsRS8, transcriptional regulation

## Abstract

Raffinose synthetase (RS) is a key enzyme in the process of raffinose (Raf) synthesis and is involved in plant development and stress responses through regulating Raf content. As a sweetener, Raf makes an important contribution to the sweet taste of white tea. However, studies on the identification, analysis and transcriptional regulation of *CsRSs* (*Camellia sinensis* RS genes) are still lacking. In this study, nine CsRSs were identified from the tea plant (*Camellia sinensis*) genome database. The CsRSs were classified into five groups in the phylogenetic tree. Expression level analysis showed that the *CsRSs* varied in different parts of the tea plant. Transcriptome data showed that *CsRSs* could respond to persistent drought and cold acclimation. Except for *CsRS5* and *CsRS9*, the expression pattern of all *CsRSs* increased at 12 h and decreased at 30 h during the withering process of white tea, consistent with the change trend of the Raf content. Furthermore, combining yeast one-hybrid assays with expression analysis, we found that CsDBB could potentially regulate the expression of *CsRS8*. Our results provide a new perspective for further research into the characterization of *CsRS* genes and the formation of the white tea flavour.

## 1. Introduction

Raffinose (Raf) is an oligosaccharide of the raffinose family and is widely present in plants [1]. The first step of Raf biosynthesis is to use UDP-galactose (UDP-Gal) and L-myo-inositol as substrates to produce galactinol (Gol, 1-O-α-D-galactopyranosyl-L-myo-inositol) under the catalysis of galactitol synthase (GolS, EC 2.4.1.123) [2]. Next, the galactosyl from myo-inositol galactinol is connected with sucrose (Suc) through α-1 and 6-glycosidic bonds to generate Raf, which is catalyzed by raffinose synthase (RS, EC 2.4.1.82) [3]. Notably, the reaction process is reversible [4]. Current studies show that Raf mostly accumulates in plant seeds, which can promote the process of cytoplasmic vitrification [5]. In addition, Raf can participate in the protection of the cell membrane by inhibiting the crystallization of sucrose during seed dehydration, improving seed storage tolerance and maintaining seed vitality [6,7,8]. Recent studies have found that Raf can also accumulate in leaves and roots as a response mechanism to abiotic stress and protect plant growth. For example, the content of Raf in *maize* leaves significantly increases under salt stress and high temperature stress [9]; the content of Raf in *Arabidopsis* shows a positive correlation with its freezing tolerance [10]; the Raf in the cytoplasm is transported to the chloroplast to protect the chloroplast structure in response to cold stress [11]; moreover, as a scavenger of ROS in plant cells, Raf can reduce the damage caused by the ROS oxidative stress reaction [12]. In the food industry, Raf is widely used as a food-grade sweet additive. Although Raf cannot be absorbed by humans, it can be used by beneficial bacteria (Bifidobacterium and Lactobacillus) in the intestine and inhibit the growth of harmful bacteria [13,14,15].

RS is a key enzyme that catalyzes the formation of Raf, and the RS gene family has been identified and characterized in many plants, including *Arabidopsis* [3], corn [16], lentil [17], cotton [18] and poplar [19]. The expression of RS genes can be induced by plant seed development and stress; *GmRS2A* and *GmRS2B* are highly expressed in the middle of soybean seed maturity, and the content of Raf also increases [20]; the seed vigor of maize mutants with *ZmRS* knockout decreases [21]; the overexpression of *AtRS5* in *Arabidopsis* improves the seed germination rate and its seedlings’ drought resistance [22]. Abiotic stress induces the expression of the RS gene in plant leaves. For example, the expression of the RS gene in rice [23], maize [24] and melon [25] was upregulated under cold stress; under drought stress, high-temperature stress, high-salt stress and other stress treatments, the expression of *RS* was induced to varying degrees [26,27]; *AtRS5* (At5g40390) in *Arabidopsis* is the only *AtRS* member involved in Raf accumulation in leaves under abiotic stress [3]. Some transcription factors are involved in regulating the expression of *RS*, which can affect the resistance by regulating the biosynthesis of Raf. The overexpression of the *Arabidopsis* heat shock transcription factor *AtHsf2* can induce an increase in *AtRS2* expression and in the Raf content and produce transgenic plants with greater stress resistance [12]. In addition, the overexpression of *BnHsf4a* in *Arabidopsis* can also induce the expression of *AtRS2* [3]; *ZmHsf2* is a heat shock transcription factor in *maize*, which can induce the expression of *RS* and improve the heat tolerance of maize [28]; the overexpression of *OsWRKY11* in rice can increase the expression of *OsRS* and the content of Raf, resulting in improved drought resistance [29]; in addition, transcription factors such as CBF and ERF can also improve the plant’s resistance to stress by regulating the expression of *RS* [30,31].

Tea is a global consumer beverage, second only to water in consumption, and is processed from leaves of the tea plant(*Camellia sinensis*) [32]. According to different processing technologies, tea is divided into six categories (white tea, oolong tea, black tea, green tea, yellow tea and black tea) [33]. In recent years, white tea has become popular with consumers because of its health benefits and fresh, sweet taste, and its output and consumption are increasing rapidly [34]. Compared with other types of tea, the processing technology of white tea is simpler, including long-term withering and drying. Among these processes, withering is a slow process of water deficit stress in leaves. During this period, various flavor compounds in the leaves change, resulting in the sweet taste of white tea [35]. Amino acids and soluble sugars contribute to the sweet and umami taste of tea infusions, particularly theanine and maltose [36,37]. However, as an important flavor compound, Raf is also involved in the resistance physiology during withering and is involved in determining the plant’s taste and resistance to abiotic stress, but its changes in this process have not been reported. The RS gene has been identified and characterized in various plants but has rarely been reported in *Camellia sinensis* until now.

Due to its important role in taste contribution and the abiotic stress response, this paper focuses on the whole genome analysis of the *CsRS* family in *Camellia sinensis* and compares it with the RS of other plants, including the tea genome database comparison, phylogenetic tree analysis, chromosome distribution and gene structure. The expression pattern of *CsRSs* and the change in Raf content during the withering process of white tea were analyzed using transcriptome data from the TPIA platform, qRT-PCR and ultrahigh-performance liquid chromatography (UHPLC). In addition, the regulator of *CsRS8* was screened out by yeast one-hybrid assays. These results help us better understand the CsRS genes and the changes in chemical compounds during the withering process of white tea and provide a new perspective for the characterization of RS in *Camellia sinensis* in the future.

## 2. Results

### 2.1. Identification of RS Family Genes in Camellia sinensis

We identified nine RS genes from the *Camellia sinensis* genome, named based on the chromosome ID, from CsRS1 to CsRS9. The CsRS genes exhibited significant variations in sequence length, protein size and physicochemical properties, with the sequence lengths ranging from 1944 bp to 2445 bp, the protein sizes ranging from 647 aa (CsRS2) to 814 aa (CsRS9), and the molecular weights (MWs) ranging from 71.63 kDa (CsRS2) to 89.28 kDa (CsRS9). The prediction results of subcellular localization showed that eight of the nine CsRSs were located in the chloroplast or cytoplasm, and only one(CsRS2) was located in the nucleus. Table S2 shows the details, including protein ID, protein length, molecular weight, isoelectric point, instability index, aliphatic index, grand average of hydropathicity, and subcellular localization.

### 2.2. Phylogenetic Analysis of the CsRS Family

**Figure 1:**
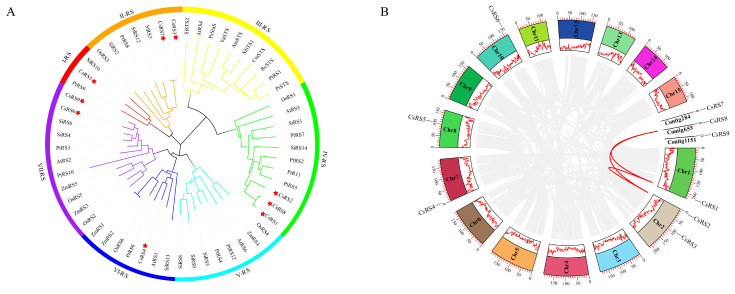
The phylogenetic tree, distribution and duplication events of CsRs. (**A**) Phylogenetic tree of RS proteins constructed using the neighbor-joining(NJ) phylogenetic tree by MEGA X. In total, 57 RS protein sequences from different species were used to construct the phylogenetic tree by MEGA X. The seven different groups are indicated by different colors. Am: *Alonsoa meridionalis*, Cm: *Cucumis melo*, Cs: *Camellia sinensis*, Os: *Oryza sativa*, Ps: *Pisum sativum*, Pt: *Populus trichocarpa*, Rc: *Ricinus communis*, Si: *Sesamum indicum*, Va: *Vigna angularis*, Vv: *Vitis vinifera*, Zm: *Zea mays*. The red star represents the RSs in *Camellis sinensis*. (**B**) Chromosomal localization and gene duplication events in CsRSs. From outside to inside, the first circle is the chromosome number with the scale on top indicating the coordinate position of the chromosome, and the second circle is the chromosome number and density. Red line indicates gene duplication.

Based on the phylogenetic tree, all RS members can be categorized into seven different clusters (I-RS, II-RS, III-RS, IV-RS, V-RS, VI-RS, and VII-RS) (Figure 1A). IV-RS and VII are the largest clusters, both with 11 members, and I-RS has only three members. AtRS5 is a protein induced by abiotic stress and involved in the biosynthesis of Raf [3]. Three CsRS members (CsRS1, CsRS2 and CsRS8) were clustered on cluster IV-RS with AtRS5. None of the CsRSs were classified as III-RS or V-RS.

### 2.3. Chromosome Location and Synteny Analysis of CsRSs

To confirm the CsRSs genomic distribution, we mapped them to the published tea plant genome (Figure 1B). *CsRSs* were unevenly distributed on chromosomes, and nine *CsRSs* were distributed on five chromosomes and three contigs. One *CsRS* was distributed on chromosome 1, chromosome 7, chromosome 8, chromosome 11, contig 284, contig 655 and contig 1151, and two *CsRSs* were distributed on chromosome 2. The Ka/Ks ratio was used to examine whether segment duplication was influenced by selection pressure during evolution. The Ka/Ks ratio of CsRS1/CsRS2 was 0.099, the Ka/Ks ratio of CsRS1/CsRS8 was 0.115, and the Ka/Ks ratio of CsRS2/CsRS8 was 0.85. The Ka/Ks ratio for all paired genes was less than 1.

### 2.4. Gene Structure and Conserved Motifs of CsRSs

**Figure 2:**
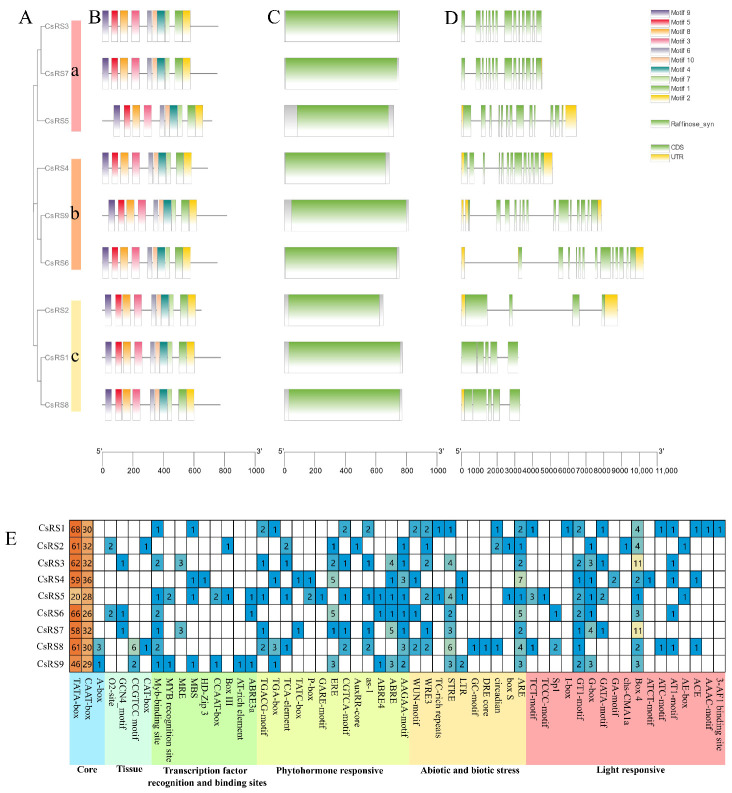
The molecular structure and cis-acting element analyses of CsRSs. (**A**) Phylogenetic analysis of RS genes in *Camellia sinensis*. CsRSs are distributed in the three subgroups on the left. (**B**) Conserved motif of CsRSs. 10 motifs are predicted by MEME and different colored boxes represent different motifs. The box lengths represent motif lengths. (**C**) Conserved domain structure of CsRSs. The green box represents the RS domain. (**D**) Exon-intron structures of CsRSs. The green boxes, yellow boxes and black lines represent the UTRs, exons and introns, respectively. (**E**) Heat map of cis-acting element of CsRSs. Different color blocks represent different promoter types.

To classify the nine CsRSs, a phylogenetic tree was constructed using MEGA X [38] based on the sequences. The nine CsRSs were divided into three classes: CsRS3/CsRS5/CsRS7, CsRS4/CsRS6/CsRS9, and CsRS1/CsRS2/CsRS8 (Figure 2A). The prediction results of the conservative motif showed that all CsRS contained 10 motifs, ranging from 6 to 50 amino acids (Figure 2B). All the identified CsRSs contain RS protein domain (Figure 2C). The number of exons per gene ranged from 4 to 14 (Figure 2D), and the genes in the same cluster showed highly similar exon numbers: 3 members (CsRS3, CsRS5 and CsRS7) in Cluster a contained 12 to 14 exons, 3 members in Cluster b (CsRS4, CsRS6 and CsRS9) contained 13 to 14 exons, and 3 members (CsRS1, CsRS2 and CsRS8) in Cluster c contained 4 to 5 exons. Significantly, CsRS1 and CsRS3 had no introns.

### 2.5. Secondary and Three-Dimensional Structures of CsRS

The predicted secondary structure of the CsRSs mainly includes random coils, α-helices and extended chains (Table 1). Swiss was used to predict the tertiary structure of the CsRSs, and the template coverage of the predicted CsRS structure was more than 99%. The predicted conformation of CsRS proteins was not identical, as shown in Figure S1A. Ramachandran’s online evaluation of protein structure showed that the amino acid sites within the allowable range were 98.5~98.8%, indicating that the constructed tertiary structure model of CsRSs was accurate and reliable (Figure S1B).

### 2.6. Cis-Elements in the Promoters of CsRSs

Fifty-six *cis*-element components were identified, with the number of *cis*-element components ranging from 87 (*CsRS5*) to 142 (*CsRS8*) (Figure 2E). Six types of *cis*-elements are included, namely light responsive, abiotic and biotic stress, phytohormone responsive, transcription factor recognition and binding sites, tissue, and core. As shown in Figure 2B, TATA and CAAT boxes were found in all nine *CsRS* promoter regions, and the A-box was specifically found in *CsRS8* and *CsRS9*. Seventeen different types of cis-elements correlated with the light response were found in the promoter region of *CsRSs*, and most *CsRSs* had Box 4, G-box and GT1-motif elements. The promoter regions of *CsRSs* also contained the *cis*-elements of phytohormone responsiveness, including abscisic acid responsiveness (AAGAA-motif and ABRE), MeJA responsiveness (CGTCA-motif and TGACG-motif), and ethylene responsiveness (ERE). Some *cis*-elements involved in abiotic/biotic stress responses and tissue-specific expression were also included: anaerobic induction (ARE), which was found in all CsRS promoter regions, and stress responsiveness (STRE), coercion responsiveness (WRE3), injury-related elements (WUN-motif) and meristem expression (CAT-box and CCGTTC-motif), which were irregularly distributed in *CsRS* promoter regions. Specifically, we found nine types of *cis*-elements of transcription factor recognition and binding sites. The myb-binding site element was present in *CsRS* promoter regions, except *CsRS2* and *CsRS4*, myb and protein binding sites (ABRE3, AT-rich element, Box III, CCAAT-box, HD-zip 3, MBS and MES) with sporadic distribution. Only *CsRS5* and *CsRS9* had the MYB recognition site element.

### 2.7. Expression Patterns of CsRSs in Different Organs

**Figure 3:**
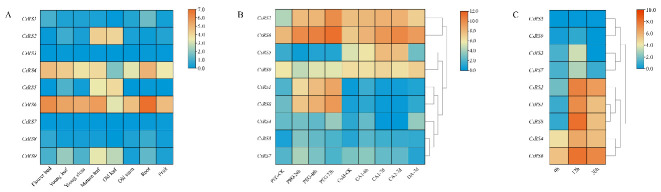
The heat map of CsRSs expression mode. (**A**) Expression pattern of CsRSs in different parts of the tea plant. (**B**) Expression pattern of CsRSs during continuous drought stress and cold acclimation. The FPKM of the continuous drought stress and cold acclimation converted by log2. PEG-CK: untreated, PEG-24: PEG treatment for 24 h, PEG-48: PEG treatment for 48 h, PEG-72: PEG treatment for 72 h, Cold-CK: nonacclimated at 25~20 °C, CA1-6 h: fully acclimated at 10 °C for 6 h, CA1-7 d: fully acclimated at 10~4 °C for 7 days, DA-7 d: recovering under 25~20 °C for 7 days. (**C**) Expression pattern of CsRSs in the withering process of white tea.

All *CsRS* genes were differentially expressed among different tissues (Figure 3A). The transcript levels of *CsRS1*, *CsRS3*, *CsRS7* and *CsRS8* were lower than those of the other members in all tissues. The transcript levels of *CsRS2*, *CsRS5* and *CsRS9* were high in mature and old leaves. Interestingly, the transcript levels of *CsRS4* and *CsRS6* were high in all tissues, especially in roots and flowers, followed by young leaves and fruits.

### 2.8. Expression Patterns of CsRSs during Continuous Drought Stress and Cold Acclimation

To investigate the expression patterns of *CsRSs* under cold acclimation and persistent drought, we downloaded and analyzed previous transcriptome data from the TPIA platform [39,40]. As shown in Figure 3B, with the increase of drought stress treatment time, all members of cluster IV-RS (*CsRS1*, *CsRS2*, and *CsRS8*) and VII-RS (*CsRS6* and *CsRS9*) were continuously upregulated; members of cluster II were upregulated at 24 h, and downregulated at 48 h and 72 h. Under cold acclimation (CA1-6 h, CA1-7 d and CA2-7 d), the expression of *CsRS2*, *CsRS5* and *CsRS6* were upregulated, and downregulated at DA-7 d.

### 2.9. Expression Patterns of CsRSs and the Raf Content in the Withering Process of White Tea

**Figure 4:**
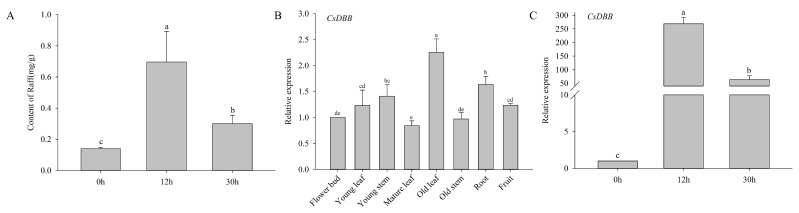
Raffinose content and *CsDBB* expression pattern. (**A**) Changes of Raf content in white tea during withering. (**B**) Expression pattern of *CsDBB* in different parts of the tea plant. (**C**) Expression pattern of *CsDBB* in the withering process of white tea. Different letters represent significant differences (*p* < 0.05).

During the withering process, the expression levels of *CsRSs* were upregulated at 12 h and downregulated at 30 h, except *CsRS5* and *CsRS6* (Figure 3C). There was no significant difference in the expression level of *CsRS5* at 0 h and 12 h, but it was significantly upregulated at 30 h. Among all *CsRSs*, the expression level of *CsRS8* peaked at 12 h, was approximately 366.5 times higher than at 0 h, and decreased at 30 h. Correspondingly, we detected the Raf content during the withering process and found that its content increased significantly at 12 hours and decreased at 30 hours, but it was still higher than at 0 h (Figure 4A). Correlation analysis showed that there was a significant positive correlation between Raf content and the expression of *CsRS1* and *CsRS8*, and a strong correlation between Raf content and *CsRS2*, *CsRS3*, *CsRS4*, *CsRS6* and *CsRS7* was found (Figure S2).

### 2.10. Screening out Transcription Factors Regulating CsRS8

The cDNA library was transformed into a bait yeast strain and inoculated on SD/-Leu + 500 ng/mL AbA medium for screening, and one transcription factor (CsDBB) was identified, which may directly bind to the *CsRS8* promoter (Figure S3A). We predicted the subcellular localization of CsDBB, and both the Wolf and Cell-PLoc results showed that CsDBB localized in the nucleus and had typical transcription factor characteristics. According to the conserved domain analysis, we found that CsDBB contains a double B-box zinc finger protein structure, which is a typical feature of DBB transcription factors (Figure S3B). The qRT-PCR results showed that *CsDBB* was expressed in different tissues (Figure 4B). During the withering process, the expression level of *CsDBB* first increased and then decreased (Figure 4C). Furthermore, the correlation analysis showed that *CsDBB* was positively correlated with *CsRS8* at the expression level and positively correlated with the Raf content (Figure S3C).

### 2.11. The Interaction and Regulation Network of CsRSs

**Figure 5:**
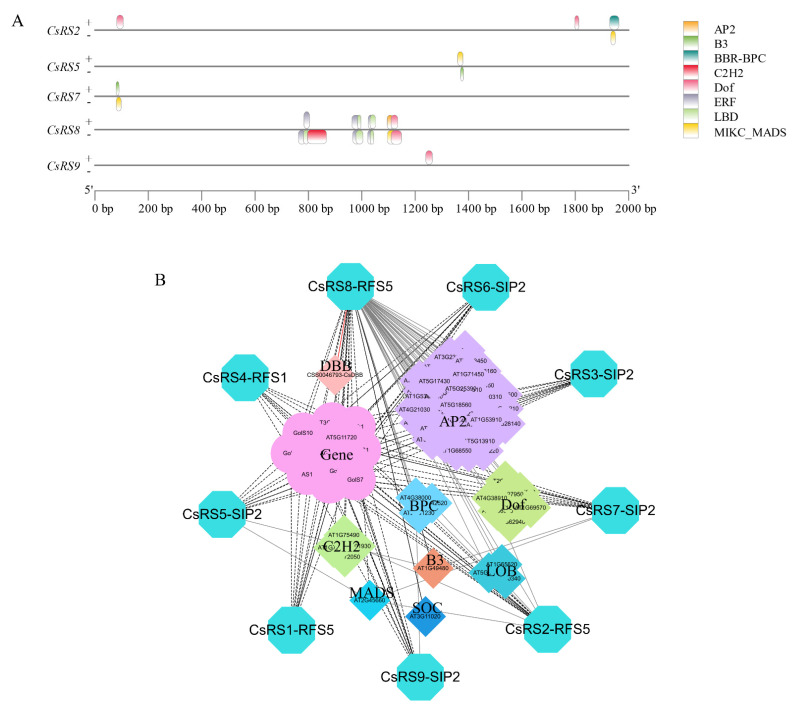
Transcription factor binding site prediction and protein interaction regulation network of CsRSs. (**A**) Transcription factor binding sites predicted in the promoters of CsRSs. Protein Interaction Regulatory Network of CsRSs. Boxes of different colors represent different transcription factor families. “+” and “−” represent positive and negative strands, respectively. Dotted lines indicate protein interactions, solid lines indicate that transcription factors regulate CsRSs, and the solid red line indicates that it has been verified by experiments. (**B**) Protein interactions and regulatory networks of CsRSs. The outermost ring represents RS in *Camellia sinensis*. The circles represent functional genes and the diamonds represent transcription factors, different color blocks represent different families of transcription factors, the dotted line represents the interaction between genes, the solid line represents the regulation of genes by transcription factors, and the red line represents preliminary validation.

To investigate the regulation of the expression of *CsRSs* by transcription factors (TF), the PlantTFDB database was used to predict transcription factor binding sites on promoters. A total of 8 TF families (AP2, B3, BBR-BPC, C2H2, Dof, ERF, LBD and MIKC_MADS) were predicted can bind the CsRSs (CsRS2, CsRS5, CsRS7, CsRS8 and CsRS9) promoters, which covers 1, 1, 3, 1, 9, 44, 2 and 1 members. The ERF family had the largest number of binding sites (114), while the AP2 family had the smallest binding sites (2). The largest number and variety of TFs were identified in the promoters of *CsRS8*, but only a TF family(C2H2) was identified in CsRS9 (Figure 5A).

We obtained the protein interaction regulatory network of CsRSs through the yeast one-hybrid, STRING and PlantTFDB databases. Nine CsRSs were compared to three AtRSs in *Arabidopsis*; CsRS4 corresponded to AtRFS1, CsRS1 and CsRS2; CsRS8 corresponded to AtRFS5, CsRS3 and CsRS5, CsRS6, and CsRS7; CsRS9 corresponded to AtSIP2; a total of 14 functional genes were predicted to interact with CsRSs; 64 transcription factors were found to possibly regulate the expression of CsRSs (Figure 5B). There are 11 galactosidases and 1 stachyose synthase in 14 functional proteins, and 64 transcription factors mainly belonging to the AP2, Dof, C2H2, LOB and other transcription factor families.

## 3. Discussion

White tea is mainly produced in China, is popular with consumers all over the world and is characterized by a sweet taste [34]. The manufacturing process of white tea includes two steps, withering and drying, and withering is the key process to form the characteristics of white tea. During the withering process, leaves undergo various stresses, mainly drought. Under water deficit stress, some stress-related flavor compounds and aroma compounds increase significantly, which contribute to the unique taste of white tea [35]. Raf plays a key role in resisting abiotic stress and taste formation [12,41]. In vivo, Raf biosynthesis is catalyzed by RS, but there is a lack of relevant research in *Camellia sinensis* [3]. Therefore, we sought to identify and characterize Raf in *Camellia sinensis* and the changes in Raf and its related genes during the withering process of white tea. In this study, we conducted a comprehensive and systematic analysis of the RS gene family of *Camellia sinensis* and explored the expression level and Raf content during the withering period.

### 3.1. Evolution of the CsRS Gene Family

RS is a key enzyme involved in Raf biosynthesis, and RS genes have been identified in many plants, including *Arabidopsis* [3,42], cassava [43], maize [16] and sesame [44]. In this study, we identified nine CsRS genes from *Camellia sinensis* distributed on five chromosomes and three contigs. Collinearity analysis showed that *CsRS1*, *CsRS2* and *CsRS8* had gene replication events, and their Ka/Ks values were all less than 1 (Figure 1B), indicating that CsRSs mainly experienced purification selection after replication [45]. Based on the evolutionary tree, we divided the selected RSs into six clusters, and nine CsRSs were irregularly distributed in five clusters (Figure 1A). Similarly, the RS evolutionary trees constructed in sesame [44] and maize [16] were also divided into six clusters. Previous studies have reported that the raffinose synthase domain (PF05691) exists in both RS and STS (stachyose synthase) proteins, but a Gol-Raf galactosyltransferase/galactosyl hydrolase domain occurs exclusively in STS sequences [3]. In our study, the sequences containing complete RS domains were selected for phylogenetic construction, and none of the CsRSs grouped together with the STS from other species. CsRS1, CsRS2, CsRS8 and AtRS5 from *Arabidopsis* are clustered in IV-RS, and CsRS1, CsRS2 and CsRS8 also have similar gene structures, indicating that these proteins may have similar functions (Figure 1A). AtRS5 is involved in the biosynthesis of raffinose in leaves under abiotic stress, and it is the only RS gene involved in raffinose biosynthesis in *Arabidopsis* [3]. Therefore, we speculate that CsRS1, CsRS2 and CsRS8 may also participate in the accumulation of Raf in tea leaves under abiotic stress.

### 3.2. Potential Role of CsRS in Abiotic Stress and the Withering Process of White Tea

The promoter is a specific DNA sequence located upstream of the gene start codon ATG that affects the expression level of foreign genes [46]. In this study, we identified the homeopathic elements of the *CsRS* promoter region and further classified them into six categories: light response elements, stress response elements, hormone response elements, transcription factor-related elements and plant growth and development elements (Figure 2E). Our results show that in addition to numerous light response elements, there are many hormone stress response elements, including MeJA, GA, ABA, ethylene and auxin response elements, indicating that hormones may affect the expression of *RS*. Under ABA treatment, the mRNA level of *CsRS* in *Cucumis sativus* was positively correlated with the concentration of ABA treatment groups, and the Raf content also increased [47]. In addition, we found that there are numerous stress response elements, such as drought response (DRE core), pressure response (Box S, STRE, TC rich repeats) and low temperature (LTR). The overexpression of *AtRS5* increases the content of raffinose in seeds, thus improving the desiccation tolerance of seeds [22]; the overexpression of *CsRS* from *Cucumis sativus* in tobacco can promote the accumulation of Raf in leaves, and cold stress can induce the expression of *CsRS* in cucumber, thereby improving the content of Raf and cold resistance [47]; RS genes are also involved in cold stress and cold acclimation in sugar beets [25], rice [23] and tea plants [48]. Combined with previous research data [39,40], the analysis of transcriptome data during continuous drought stress and cold acclimation and the relative expression of the withering process of white tea revealed that the expression of *CsRSs* under different stresses differed. The members of the same cluster have the same expression pattern under continuous drought stress, especially the cluster of IV-RS and VII-RS that were up-regulated continuously; *CsRS2*, *CsRS5* and *CsRS6* were induced during cold acclimation. The withering is a slow process of inducing water deficit stress in the leaves; *CsRS1*, *CsRS2*, *CsRS3*, *CsRS4*, *CsRS7*, *CsRS8* and *CsRS9* were up-regulated at 12h and down-regulated at 30h, and the content of Raf has the same trend.

In conclusion, we speculate that *CsRSs* may play an important role in the accumulation of Raf in the process of tea plant stress. Most notably, *CsRS8* contains the most types and quantities of *cis*-acting elements in response to stress, which may play a more crucial role in the response to stress. The qRT-PCR results showed that most of the *CsRSs* were highly expressed at 12 h of withering, especially CsRS8 (Figure 3C), which had the most significant change, and its expression was significantly positively correlated with the content of Raf (Figure S2), so we speculate that *CsRS8* played a significant role in the accumulation of Raf during the withering process.

### 3.3. CsDBB Potentially Regulates CsRS8 to Affect Raffinose Accumulation during the Withering of White Tea

Transcription factors are special structural proteins that can regulate plant growth and development and regulate the specific expression of target genes by binding *cis*-acting elements in the promoter region of target genes [49]. Recent studies have shown that *RS* is regulated by various transcription factors. The overexpression of the heat shock transcription factors *Arabidopsis AtHSFA2* and *Brassica napus BnHSFA4a* can induce the expression of *AtRS2* [12,50]. Maize *ZmDREB2A* can regulate the expression of *RS* and prolong the preservation time of seeds [51]. In addition, WRKY, ERF and other types of transcription factors are involved in regulating the expression of RS genes [31,52]. In our study, we predicted that 59 transcription factors belonging to eight TF families (AP2, B3, BCR-BPC, C2H2, Dof, ERF, LBD and MIKC_MADS) could potentially regulate the expression of *CsRSs*. *Cis*-element prediction results showed that *CsRS8* contained the most types and quantities of elements, indicating that *CsRS8* may be regulated by multiple transcription factors (Figure 2E). Besides, six categories of transcription factors were identified, all of which could potentially regulate *CsRS8* (Figure 5A), further confirming our speculation. DBB belongs to the subfamily of b-box transcription factors and is named because it contains two b-box structures involved in plant growth and development and abiotic stress response [53]. In our study, a CsDBB family transcription factor, CsDBB (CSS0046793), was screened by yeast one-hybrid assays (Figure S3A). We found that *CsDBB* was significantly upregulated and then decreased during the withering process (Figure 4B). The expression level of *CsDBB* was positively correlated with *CsRS8*, *CsDBB* and Raf content, according to the correlation analysis results (Figure S3C). Therefore, we speculated that CsDBB might positively regulate *CsRS8* during the withering process, affecting the accumulation of Raf. We predicted the protein interaction regulation network of CsRSs and found that there are nine functional proteins of the carbohydrate metabolism pathway that have potential interactions with CsRS (Figure 5). We found several transcription factor families, such as AP2, Dof and C2H2, indicating that there is a complex protein interaction regulation network in the carbohydrate metabolism pathway that regulates sugar components in the stress environment to resist external stress.

## 4. Materials and Methods

### 4.1. Identification of CsRSs from Tea Genome

The genome database of tea cultivar “Shuchazao” (Camellia sinensis) was used in this study [54]. The identification of RS genes was carried out with HMMsearch using PF05691 as the Pfam domains, which were downloaded from the Pfam database (http://pfam.xfam.org, accessed on 24 December 2021) [55], and the HMMsearch was performed by SPDE v1.2 [56]. To verify the presence of the significant RS domain, all the sequences were examined using the Pfam database [55] and NCBI CDD database (https://www.ncbi.nlm.nih.gov/Structure/cdd/wrpsb.cgi, accessed on 24 December 2021) [57]. The protein sequence, coding sequence, promoters and gene annotation were downloaded from the Tea Plant Information Archive (TPIA) (http://tpdb.shengxin.ren/index.html, accessed on 24 December 2021) [39]. The chromosome localization, collinearity and gene duplications events were analyzed using TBtools software [58]. Tools at the ProtParam in ExPASy web (https://web.expasy.org/protparam/, accessed on 24 December 2021) [59] and WoLF PSORT (https://wolfpsort.hgc.jp/, accessed on 24 December 2021) [60] were used to predict the physicochemical characteristics and the subcellular localization of the identified CsRS proteins. 

### 4.2. Phylogenetic Tree Construction and Structural Analysis of CsRSs

To clarify the phylogenetic relationship of the CsRS genes, a multi-sequence alignment was constructed with the MEGA X software [38] with the neighbor-joining (NJ) algorithm with 2000 bootstrap replicates, and the phylogenetic tree was refined by the iTOL tool (https://itol.embl.de/, accessed on 29 January 2022) [61]. The exon/intron structures of the CsRSs were determined by TBtools software [58]. The secondary and three-dimensional structures of the CsRS were obtained by homology modeling using the SOMPA (https://npsa-prabi.ibcp.fr/cgi-bin/npsa_automat.pl?page=npsa_sopma.html, accessed on 25 December 2021) and SWISS-MODEL (https://swissmodel.expasy.org/, accessed on 21 September 2022) [62]. The CsRSs protein tertiary structure model of CsRSs was evaluated using the PROCHECK plugin in the PDBsum online database (http://www.ebi.ac.uk/thorntonsrv/databases/cgibin/pdbsum/GetPage.pl?pdbcode=index.html, accessed on 21 September 2022) [63,64].

### 4.3. Cis-Element Prediction for CsRSs Promoters

The promoter sequences (2 kb upstream of ATG) of CsRSs were exported by TBtools software [58], then all the promoter sequences were submitted to the PlantCARE database (http://bioinformatics.psb.ugent.be/webtools/plantcare/html/, accessed on 1 February 2022) [65], and TBtools software [58] was used for visualization.

### 4.4. Plant Materials and Treatments

Samples for relative expression pattern analysis: a total of eight tissue samples (young leaf, mature leaf, old leaf, young stem, old stem, flower bud, seed, and root) were collected from Camellia sinensis cultivar “Huangdan” (HD) growing in Fujian Agriculture and Forestry University, Fuzhou, China (26°05′ N, 119°18′ E), in September 2020. Camellia sinensis cultivar “Fudingdahao” (FDDH) is a kind of tea variety suitable for white tea that is widely planted in southern China. Processing sample: the young shoots with one bud and two leaves were harvested from the FDDH for making white tea. The fresh leaves were processed according to the standard white tea production procedure, including wilting and drying. During the withering period, sampling occurred at 0 h, 12 h and 30 h (3 times), with 0 h as control, and the whole process was carried out in an air-conditioned room (relative humidity 70 ± 3%, temperature 37 ± 3 °C). All samples were immersed in liquid nitrogen immediately after collection, and then stored at −80 °C. Three replicates were collected for each treatment. All samples for chemical compositions and gene expression analyses were extracted and analyzed in triplicate.

### 4.5. Analysis of Raffinose Content in the Withering Process of White Tea

The contents of Raf were determined by ultra-high performance liquid chromatography (UHPLC) (Waters, Milford, MA, USA). All samples from the withering process of white tea (0 h, 12 h and 30 h) were dried in a vacuum freeze-dryer. A total of 0.25 g of dried and fully crushed tea samples was added to distilled water (2.5 mL, 100 °C), soaked in a 100 °C water bath for 15 min, stirred 2–3 times (every 5 min), and centrifuged to obtain the supernatant. The supernatant was transferred to a 25 mL volumetric flask, and repeated extraction once. The supernatant obtained from two extractions was combined, 15mL acetonitrile was added, and added distilled water to the total volume of 25 mL. Samples were then filtered through a 0.22 µm membrane before analysis. Chromatographic conditions were set as previous studies with minor modifications and an ACQUITY UPLC BEH Amide column (Waters, Milford, MA, USA) was used to separate Raf [66].

### 4.6. Analysis of CsRSs Expression Patterns

The expression patterns of *CsRSs* during cold acclimation and continuous drought stress were analyzed using the previous transcriptome date downloaded from the TPIA platform [39,40]. The fragments per kilobase of exon model per million mapped fragments (FPKM) values of *CsRS* genes were extracted from the transcriptome date, and the heatmap was drawn using TBtools software [56]. The qRT-PCR results were used to analyze the expression patterns of *CsRSs* in different parts of the tea plant and during the withering process of white tea. Total RNA was obtained from samples using a RNAprep Pure Plant Plus Kit (Tiangen, Beijing, China) according to manufacturer instructions. RNA samples were reverse-transcribed into cDNA using One-Step gDNA Removal (TransGen, Beijing, China), and the synthesis reaction was carried out following the manufacturer protocol. The qRT-PCR analysis was carried out on qTOWER^3^G (Analytik Jena AG, Jena, Germany) with a 20 μL reaction mixture, with the following thermal parameters: 95 °C for 30 s, followed by 40 cycles of 95 °C for 5 s and 60 °C for 30 s. A melt curve was performed to verify product specificity of the PCR at the end of each reaction. Relative gene transcript levels were calculated by the 2^−ΔΔct^ [67]. Gene expression values were log_2_ transformed, and heatmaps were drafted by TBtools software [58]. The gene-specific primers are listed in Table S1. Changes in mRNA levels of related genes were normalized to that of *CsGAPDH*.

### 4.7. Yeast One-Hybrid Screeing

Combined with RS phylogenetic tree and correlation analysis, the 500 bp promoter sequence of the *CsRS8* was inserted into the pAbAi vector to construct the bait vector pAbAi-CsRS8 promoter. Then, the bait vector was integrated into the Y1HGold yeast strain by the PEG/LiAc transformation method to form the Y1H-pAbAi-CsRS8 promoter bait yeast strain. The bait yeast strain was inoculated on SD/-Ura medium and SD/-Ura + 100/150/300/500 ng/ml Aureobasidin A(AbA) medium to determine the minimum amount of AbA to inhibit the basal expression level of the bait yeast strain inhibitory concentration. cDNA library plasmids were purchased from Nanjing Ruiyuan Biotechnology Co., Ltd. The cDNA library was transformed into the Y1H-pAbAi-CsRS8 promoter bait yeast strain and inoculated on SD/-Leu + 500 ng/mL AbA medium for selection. The plasmid was extracted from the positive yeast monoclonal, transferred to Y1H-pAbAi-CsRS8 promoter bait yeast strain, and spotted on SD/-Leu medium and SD/-Leu + 500 ng/mL AbA medium. After 2 days of incubation at 30 °C, the interaction between CsDBB and the bait sequence was observed.

### 4.8. Prediction of CsRSS Protein Interactions and Regulatory Networks

STRING (https://cn.string-db.org/, accessed on 1 October 2022) and PlantTFDB (http://planttfdb.gao-lab.org/, accessed on 5 October 2022) were used to predict the protein interaction regulatory network of CsRS. In the STRING database, we input the CsRSs protein sequence and searched the protein interaction network with Arabidopsis as a reference. In the plantTFDB database, we input the CsRSs promoter region (2000 bp before ATG) and set the threshold *p*-value < 1 × 10^−7^ to predict the transcription factor binding sites and find the upstream regulators. Visualization of transcription factor binding sites was performed using TBtools software [58]. The network diagram of protein interaction and regulation was drawn using the Cytoscape 22.0 software.

### 4.9. Statistical Analyses

Data analysis was performed using Excel (2020) and SPSS (Version 26, SPSS Inc., Chicago, IL, USA). One-way analysis of variance (ANOVA) was performed, followed by Duncan’s multiple range test at *p* < 0.05.

## 5. Conclusions

In this study, nine CsRSs were identified and analyzed. The analysis of phylogenetic relationships, conserved motifs, chromosome locations, gene structures, cis-elements and expression patterns showed that CsRSs are involved in the development process and stress response of tea plants. CsRSs are able to respond to continuous drought stress and cold acclimation. In addition, CsRSs participated in the accumulation of Raf during the withering process of white tea, especially CsRS1 and CsRS8, which had a significant correlation with the accumulation of Raf, and CsDBB might regulate the expression of CsRS8 during the withering process of white tea. This study can provide a reference for exploring the function of CsRSs and studying the quality formation mechanism during the withering process of white tea.

## Figures and Tables

**Table 1:** Secondary structure of CsRSs protein.

Gene ID	Alpha Helix	Extended Strand	Beta Turn	Random Coil
CsRS1	28.33%	21.73%	6.86%	43.08%
CsRS2	32.77%	16.85%	6.34%	44.05%
CsRS3	27.87%	21.53%	7%	43.59%
CsRS4	28.92%	19.62%	6.25%	45.20%
CsRS5	30.68%	18.41%	5.44%	45.47%
CsRS6	26.99%	21.68%	7.31%	44.02%
CsRS7	28.36%	21.84%	8.52%	41.28%
CsRS8	27.89%	20.62%	6.23%	45.27%
CsRS9	29.73%	21.62%	7%	41.65%

## Data Availability

Not applicable.

## Supplementary Materials

The following supporting information can be downloaded at: https://www.mdpi.com/article/10.3390/ijms24010202/s1.
