# Supplementary material for: Identification, Characterization and Expression Profiling of the RS Gene Family during the Withering Process of White Tea in the Tea Plant (Camellia sinensis) Reveal the Transcriptional Regulation of CsRS8
Source: Int J Mol Sci. 2022 Dec 22;24(1):202. doi: 10.3390/ijms24010202 (PMC9820808; doi:10.3390/ijms24010202)
Supplement: Supplementary file 1 [file ijms-24-00202-s001.zip › Annotations of supplementary figures.pdf]

Figure S1: The tertiary structure of CsRSs protein predicted by SWISS-MODEL; (A) Tertiary structure of CsRSs predicted by SWISS-MODEL; (B) Evaluation of the tertiary structure model of CsRSs.

Figure S2: Correlation analysis between *CsRSs* expression and Raf content in withering process of white tea.

Figure S3: The results of Y1H and correlation analysis. (A) Results of yeast one-hybrid for CsRS8. (B) Protein domain analysis of CsDBB (CSS0046793). (C) Correlation analysis between the expression levels of CsDBB and CsRS8 and raffinose content in white tea withering process.
