# Supplementary figures and images for: Identification, Characterization and Expression Profiling of the RS Gene Family during the Withering Process of White Tea in the Tea Plant (Camellia sinensis) Reveal the Transcriptional Regulation of CsRS8
Source: Int J Mol Sci. 2022 Dec 22;24(1):202. doi: 10.3390/ijms24010202 (PMC9820808; doi:10.3390/ijms24010202)

A

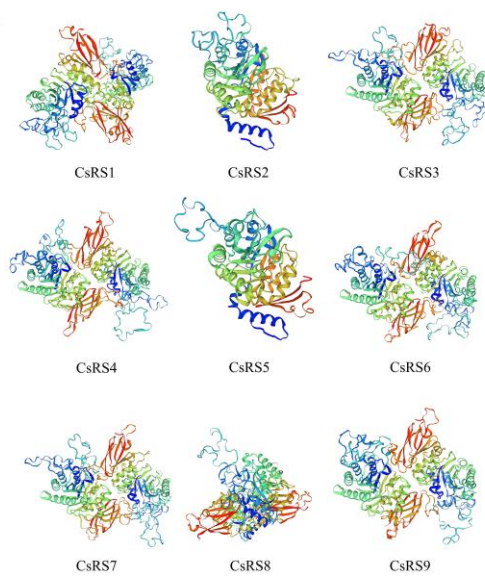

B

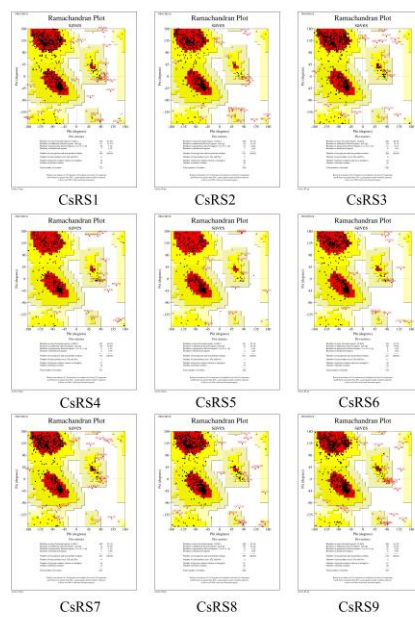

Supplement: Supplementary file 1 [file ijms-24-00202-s001.zip › Figure S1-Tertiary structure and quality evaluation of CsRSs protein.pdf]

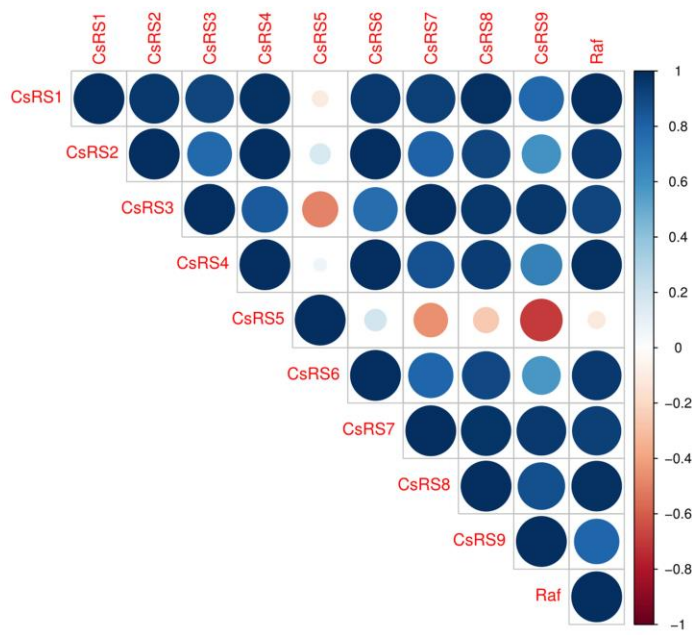

Supplement: Supplementary file 1 [file ijms-24-00202-s001.zip › Figure S2 Correlation analysis between CsRSs expression and Raf content in withering process of white tea.pdf]

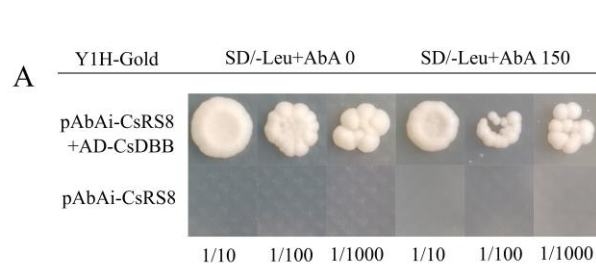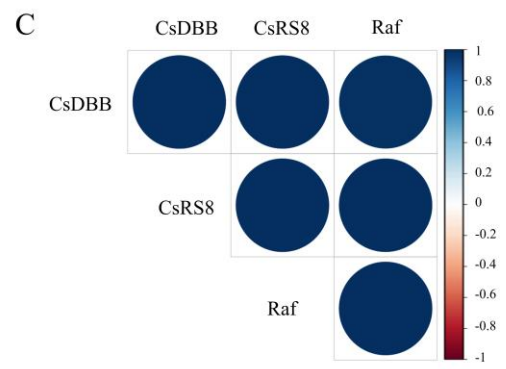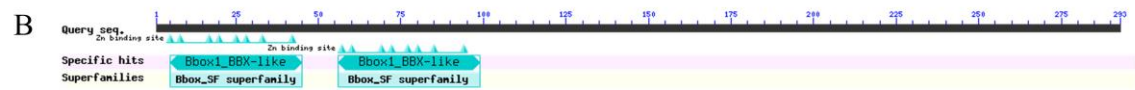

Supplement: Supplementary file 1 [file ijms-24-00202-s001.zip › Figure S3-The results of Y1H and correlation analysis.pdf]
